# Remarks on the Efficacy of Cold Affusion in Typhus and Scarlet Fever

**Published:** 1815-05

**Authors:** Thomas Lodge

**Affiliations:** Surgeon, Preston, Lancashire.


### *For the Medical and Physical Journal.*

### *Remarks on the Efficacy of Cold Affusion in Typhus and Scarlet Fever;* by Mr. Thomas Lodge, Surgeon, Preston, Lancashire.

WHEN I reflect on the great mortality from *typhus* and *scarlatina*, and call to my recollection the methods of treatment generally employed in those fevers, at least in this this large and populous county, I cannot refrain from attributing the failure of the treatment, in the majority of the fatal cases, to the omission of that invaluable (I had nearly said specific) remedy the *cold affusion*, which, I am sorry to observe, is by no means generally employed, much less recommended, by medical practitioners. During the last three years, I have had an opportunity of witnessing its happy effects in some hundreds of cases of typhus and scarlatina, and I trust your intelligent readers, especially those who have perused Dr. Currie's invaluable book on the subject, will give me credit when I say, that I never witnessed a single instance of death from either of the fevers in question, when the affusion, employed in the manner recommended by Dr. Currie, had not been postponed beyond the 3d day of the attack: nay, if used at a latter period, viz. from the 5th to the 9th day, it will generally, I have observed, render the symptoms milder, and occasionally cut short the disease. Hence, I conceive, that if a medical practitioner be consulted in a case of incipient typhus or scarlatina, and he omit to recommend the cold affusion, he has not done the patient justice; he has not done his duty; because he has neglected to recommend the remedy which, of all others, would have been most likely to have cured his patient.

Sponging the body with vinegar has been recommended by Dr. Currie and others, in typhus and scarlatina. However, I feel it my duty to state, that, in the cases where I have had an opportunity of seeing it employed, it invariably appeared to me to do harm. In a few minutes after its employment, the patient feels stiff and uncomfortable, from the mucilaginous matter contained in the vinegar clogging up the pores of the skin, and thereby preventing evaporation; on which principle, probably, the extraordinary efficacy of the effusion and sponging with cold water in a great measure depend.

*April* 6, 1815. THOMAS LODGE.

---

*For the Medical and Physical Journal.*

